# Nordic Innovative Trial to Evaluate OsteoPorotic Fractures (NITEP-group): non-operative treatment versus surgery with volar locking plate in the treatment of distal radius fracture in patients aged 65 and over – a study protocol for a prospective, randomized controlled trial

**DOI:** 10.1186/s12891-018-2019-5

**Published:** 2018-04-05

**Authors:** Teemu P. Hevonkorpi, Antti P. Launonen, Lauri Raittio, Toni Luokkala, Juha Kukkonen, Aleksi Reito, Bakir O. Sumrein, Minna K. Laitinen, Ville M. Mattila, on behalf of the NITEP-group

**Affiliations:** 1 School of Medicine, University of Tampere, 33014 Tampere, Finland; 2 Department of Orthopaedics, Unit of Musculoskeletal Surgery, Tampere University Hospital, Teiskontie 35, PL2000, 33521 Tampere, Finland; 3 Central Finland Central Hospital, Keskussairaalantie 19, 40620 Jyväskylä, Finland; 4 Satakunta Central Hospital, Sairaalantie 3, 28500 Pori, Finland

**Keywords:** Distal radius, Operative treatment, Non-operative treatment, Volar plating, Forearm fractures, Randomized controlled trial, RCT

## Abstract

**Background:**

In the literature, there are numerous studies that compare different surgical procedures in the treatment of distal radius fractures (DRF). It is, however, unknown whether operative treatment and better restoration of anatomy with volar locking plate yields a better functional outcome in the elderly population when compared with non-operative treatment.

**Methods and design:**

This study is a prospective, randomized, controlled, multi-center trial. The purpose will be to compare the non-operative and operative treatment of initially or early malaligned distal radius fractures in patients aged 65 and older. The primary outcome in this study will be the patient rated wrist evaluation (PRWE) score measured after 1 and 2 years.

**Discussion:**

We expect that initial operative treatment of a DRF with volar locking plate will not yield superior results when compared with non-operative treatment with cast immobilization in terms of functional outcome, pain, disability, quality of life, grip strength, and number of complications.

**Trial registration:**

This trial is registered on clinicaltrials.gov, identifier NCT02879656, registration date 08/17/2016.

**Electronic supplementary material:**

The online version of this article (10.1186/s12891-018-2019-5) contains supplementary material, which is available to authorized users.

## Background

In the aging population, distal radius fracture (DRF) is one of the most common fractures and accounts for 4% of all fractures [1–4]. The age-adjusted overall incidence of DRF has varied between 100 to 300 per 100,000 person-years depending on sample, and it is more common (200 to 1200 per 100,000 person-years) in the elderly population [5, 6]. In Finland, the estimated annual number of DRFs among patients aged 60 and older is approximately 8000 to 9000.

In addition to the significant disability caused by DRFs among older individuals, these injuries are associated with a high economic impact. In general, the operative interventions, outpatient visits, and rehabilitation after a fracture put additional strains on scarce resources. The minimum direct cost of every operatively-treated fracture has been estimated to be approximately 1400 to 6800 € [7–9]. Considering the annual number of these fractures, it is essential that our limited resources are targeted at treatment methods with proven efficacy and cost-effectiveness.

Interestingly, some common orthopaedic operative interventions among older individuals, such as distal radius fracture surgery, are not based on well-established, high-quality scientific evidence [10]. In fact, there have been numerous studies that show the number of operative interventions for DRF is increasing, even though there is no high quality scientific evidence to support operative treatment [11, 12]. This is especially the case in patients aged over 60.

Treatment options for DRFs have varied between non-operative and operative, and numerous different surgical methods have been described over time. Operative treatment with a volar locking plate was introduced in the early 2000s, and since then the procedure has rapidly gained wide popularity. The aim of the procedure is to improve the repair of osteoporotic or comminuted fractures by providing a stable construction [12]. It has also been hypothesized that reducing the fracture to the normal anatomical position would produce a superior functional outcome [13]. Several other operative techniques, such as dorsal plates, fragment-specific plates, external fixators, metal wires and screws, have been proposed for treating DRFs. However, none of them have been shown to be superior to any other. According to several high-quality randomized controlled trials, percutaneous techniques, such as external fixator and metal wires, produced similar functional outcomes when compared with a volar locking plate [14, 15].

The most important question has, however, remained unanswered; whether operative treatment is superior to non-operative treatment in terms of functional outcome (based on Patient Rated Outcome Measures (PROMs)) and cost-efficiency in the older population. Undoubtedly, operative interventions result in fewer malunions compared with closed reduction and immobilization with a plaster cast [16]. However, non-operative treatment may be related to lower complication rates in terms of non-mechanical and mechanical problems, such as infection, wound breakage, and technical mistakes [17].

It has been shown that the functional outcome of a DRF correlates with the anatomical position of the articular surface in young, active patients. In the older population, however, the anatomic parameters correlate poorly with a positive long-term functional outcome [18, 19]. The challenge is to apply the results derived from the studies into clinical practice, especially with elderly patients. It is evident that not all people aged 65 and older are part of a homogeneous population of frail people with consistent low demands regarding physical activity. Indeed, contradicting reports have also been published regarding the correlation of anatomical parameters and functional outcome, and it has been suggested that there might be a subset of elderly patients who suffer from malunion. [20, 21] Setting a specific threshold on a continuum (i.e., age or a specific radiographic parameter) might therefore be arbitrary. The obvious challenge is to predict the subset of patients who are at risk from the majority of patients who do well.

Contrary to anatomic parameters, certain patient-related characteristics have been shown to correlate well with the outcome of the treatment [22]. These characteristics include variables, such as pain-related anxiety, catastrophic thinking related to pain, and the severity of acute pain, which can be measured by using the pain catastrophizing scale (PCS) [23]. The PCS has been used to measure trauma-related catastrophic thinking, and it has been found to associate with finger stiffness after the operative treatment of DRF, for instance [24].

Previous studies that have compared operative to non-operative treatment have had several study limitations that include small sample sizes, compromised outcome measures, and selection biases that have led to controversial conclusions about treatment [25]. Thus, the aim of this randomized prospective trial will be to compare the non-operative and operative treatment of initially or early malaligned distal radius fractures in patients aged 65 and older in terms of functional outcome.

## Methods and design

### Study design

The study will be a prospective, randomized, controlled, multi-center trial. The aim of the study will be to compare non-operative and operative treatment (open reduction and internal fixation with volar locking plate) of initially or early (5 to 10 days) malaligned distal radius fractures.

### Aims of the study

The specific aims of this trial are as follows:

(i) to compare non-operative treatment to volar plating in the treatment of initially malaligned distal radius fractures in patients aged 65 and older in terms of functional outcome measured with PRWE
(ii) to compare non-operative treatment to volar plating in the treatment of distal radius fractures with early instability during follow-up, i.e., loss of reduction at 1 week (range 5 to 10 days) in patients aged 65 and older in terms of functional outcome measured with PRWE
(iii) to compare pain, disability, quality of life, grip strength, and the number of complications after non-operative treatment and the initial and delayed operative treatment of distal radius fracture
(iv) to assess the effect of pain catastrophizing score (PCS) on the functional outcome of non-operatively and operatively treated distal radius fracture
(v) to assess the association between physical activity and the number of wrist movements measured with Axivity accelerometer and functional outcome measured with PROMs of non-operatively and operatively treated distal radius fractures
(vi) to assess the effect of initial as well as the final radiological parameters on the functional outcome
(vii) to assess the correlation of probability of radiological malalignment estimated by clinical prediction rule (EWC) with functional outcome measured with PRWE and PASS

### Primary outcome

The primary outcome will be the PRWE score measured after 1 and 2 years.

### Secondary outcomes

The secondary outcomes measured will be disability [QuickDASH (disabilities of the arm, shoulder, and hand)], pain in the visual analogue scale (VAS), PCS, physical activity and the number of wrist movements measured with Axivity accelerometer, quality of life (15-D), complications, and the number of surgical interventions in the non-operatively treated group. Subgroup analysis will be performed to find out patient specific features indicating good or poor outcome. PCS will be used to assess whether mental susceptibility has an influence on the functional outcome. The Axivity accelerometer will be used for the objective evaluation of the patient’s physical activity and movements of the fractured wrist in a subsample of patients. It will be used for 4 days at the 3-month and 1-year follow-up time points. EWC will be used to assess the probability of radiological malalignment from initial radiographs after injury, and its correlation with functional outcome and patient self-assessed state of symptom will be defined.

### Hypotheses

Our primary hypotheses are as follows:

(i) Initial operative treatment of distal radius fracture with volar locking plates does not yield superior results compared with non-operative treatment.
(ii) Late operative treatment of distal radius fracture with volar locking plates does not yield superior results compared with continued non-operative treatment.
(iii) Initial or delayed operative treatment of malaligned distal radius fracture does not result in superior results compared with non-operative treatment with regard to pain, disability, quality of life, grip strength, and number of complications.
(iv) A high pain catastrophizing score predicts a poor functional outcome on the PRWE scale.
(v) A high level of physical activity and a high number of wrist movements predict good functional outcome on the PRWE scale and correlate negatively with the PCS.
(vi) Significant initial dislocation in radiographs (and high probability of malunion in EWC results) predicts a subset of poor functional outcomes on the PRWE scale and on PASS in patients between 65 to 74 years of age but not in patients aged 75 and older.

### Study population

The eligible study population will comprise all consecutive patients aged over 64 treated for a DRF, either identified in the public or referred by the emergency rooms (ERs) of the participating hospitals (Tampere University Hospital, Finland; Central Finland Central Hospital, Finland; Satakunta Central Hospital, Finland; Viborg Regional Hospital, Denmark).

The following patient selection criteria will be used throughout the study:

Inclusion criteria:

- a low-energy, intra- or extra-articular, dorsally displaced DRF within 3 cm of the radiocarpal joint, diagnosed with lateral and posterior-anterior radiographs in ER
- > 10° dorsal tilt and/or over 2 mm step-off and/or over 3 mm ulnar variance before the closed reduction of the fracture

Exclusion criteria:

- Refusal to participate in the study
- Open fracture with a severity greater than Gustilo grade 1
- Patient aged under 65
- Chauffeur’s or Barton’s fracture
- Smith’s fracture (volar angulation of the fracture)
- Patient does not understand written or spoken guidance in local languages
- Pathological fracture or a previous fracture in the same wrist or forearm

To improve patient involvement in this trial, we will interview patients with DRF before the onset of the trial. The aim of the interviews will be to move towards patient-centered medicine by taking into account the patients’ preferences and beliefs for a good outcome. To identify the questions to ask and the outcomes to measure, we will involve patients by asking questions at the beginning of the treatment. The questionnaires that will be used for patient self-assessment are attached as additional files (consult Additional files 1 and 2). The questionnaires will be repeated after 1 year, and the difference or indifference between the primary and follow-up responses will be reported.

### Randomization

Patients will be randomized with a random number matrix in block allocation fashion. The blocks will be age, site, and intra- vs. extra-articular dependent because, according to the literature, age, the presence of an intra-articular fracture, and functional outcome are associated [26, 27]. The treatment allocations derived from the matrix will be sealed in an envelope. After patient enrolment has been confirmed and informed consent has been obtained, randomization will be performed by a Tampere University Hospital research coordinator who will not otherwise attend the study. The physician responsible for the intervention or treatment will not participate in measurements or in obtaining the questionnaires. The research coordinator will monitor the study flow. An independent monitoring committee has been established.

### Intervention

#### Phase 1

All patients visiting the ER with DRF will undergo a closed reduction under local anesthesia by means of a local infiltration of Lidocaine 1%. The technique of closed reduction will not be limited to some specific method. After reduction, radiographs will be taken to verify the position of the fracture.

**Fig. 1:**
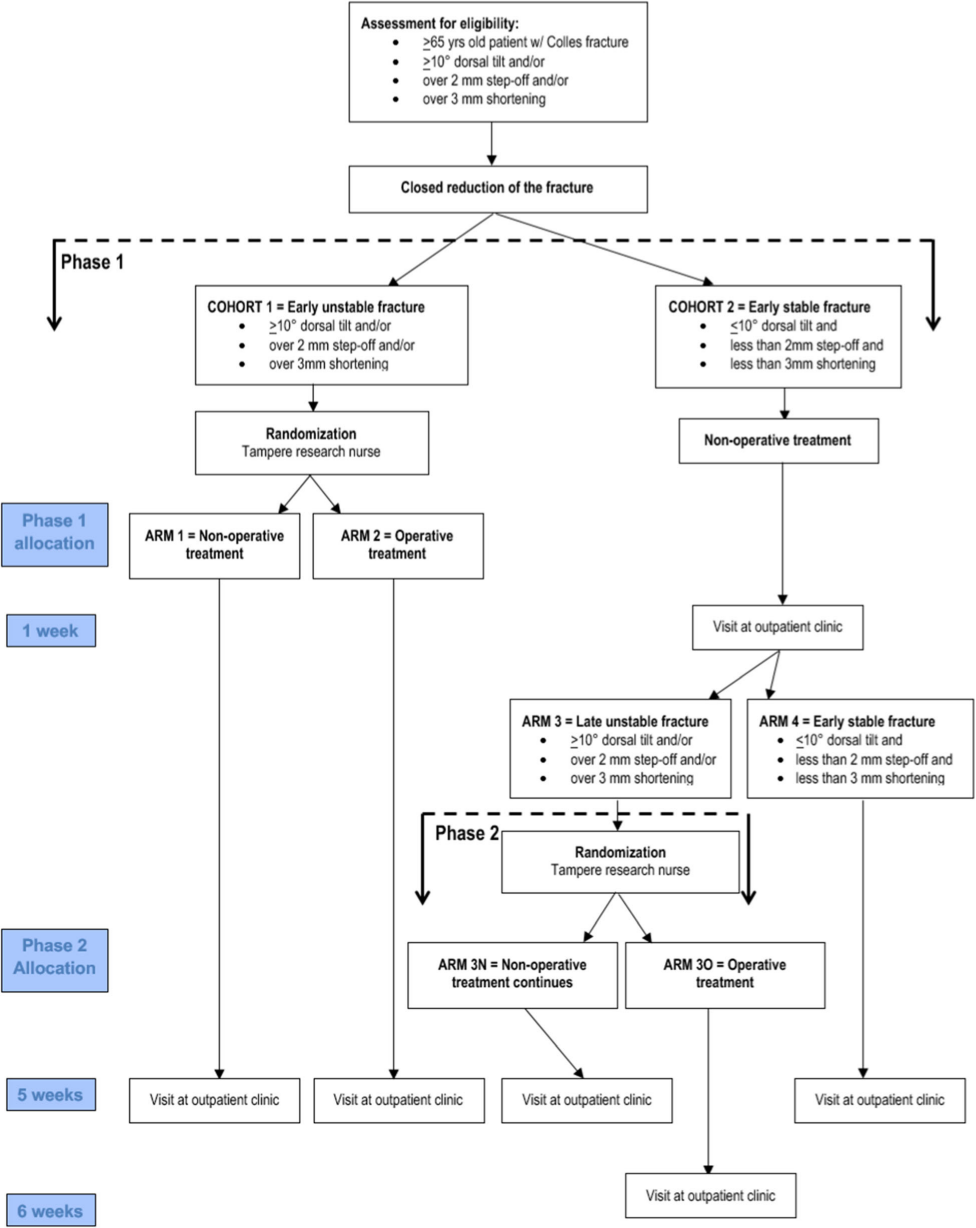
Patient flow chart of the trial

Patients will be asked to visit the study doctor at the outpatient clinic 1 to 3 days after the reduction. During the visit, patients will be asked to participate in the study. Thereafter, enrollment will be confirmed and informed consent obtained. The post-reduction radiograph will be analyzed during the visit, and patient allocation will be as follows; If satisfactory position (< 10° dorsal tilt, less than 2 mm step-off and less than 3 mm ulnar variance) after closed reduction is achieved, the patient will be allocated to Cohort 2 and non-operative treatment will be performed as usual (study flow from Fig. 1). If satisfactory reduction is not achieved (> 10° dorsal tilt and/or > 2 mm step-off and/or > 3 mm ulnar variance), the patient will be allocated to Cohort 1. After allocation, the patient will be asked to fill in the PCS, 15-D, patient history, and self-assessment questionnaires. EWC will be used to assess the probability of radiological malalignment.

In Cohort 1, the patients will be randomized to either non-operative (=Arm 1) or operative treatment (=Arm 2). Patients allocated to non-operative treatment will undergo a standard treatment protocol with a dorsal cast for 5 weeks. Patients allocated to operative treatment will undergo surgery with a volar locking plate by modified Henry’s volar approach. A dorsal cast will be used for 2 weeks. After cast removal, a physiotherapist will guide the patients in carrying out non-weight bearing, full range of motion exercises that will be continued until the 5-week follow-up appointment. After this, progressive weight bearing active and passive exercises will be conducted. From week 5 in the non-operative arm, the exercises will follow a similar exercise protocol as in the operative group.

#### Phase 2

After 1 week, the patients allocated to Cohort 2 will visit the orthopaedic outpatient clinic at the hospital where the treatment was initially started. This visit is part of the study protocol. If reduction is maintained, the patients will undergo standard follow-up visits (=Arm 4). If reduction is lost to fulfill the inclusion criteria for surgery (> 10° dorsal tilt and/or > 2 mm step-off and/or > 3 mm ulnar variance) the patient will be asked to participate to phase 2 of the study. After enrollment of the patients has been confirmed and informed consent signed, the patients will be randomized to either non-operative (=Arm 3 N) or operative treatment (=Arm 3O). If allocated to non-operative treatment, patients will undergo the same protocol as those in Arm 1. Patients allocated to operative treatment will undergo surgery with a volar locking plate by modified Henry’s volar approach as described above. In addition, physiotherapy and exercises will be conducted as described above.

Patients who decline to attend the intervention trial will be asked to join the external follow-up group. This group will be used as external validation; the group content and outcomes will be compared with the allocated intervention and control groups. The treatment of this group will be carried out in line with normal clinical practice, but the patients will have the same follow-up and asked to fill in the same questionnaires as the allocated patients.

### Follow-up

Non-operatively treated patients in Arm 1 will be treated with cast immobilization. After 5 weeks, the cast will be removed and the patients will visit the orthopaedic outpatient clinic at the hospital for follow-up visits. During the visit, direct lateral and anteroposterior radiographs will be taken. Patients will visit the outpatient clinic again after 3 months and follow-up assessment will be performed by completing the PRWE, QuickDASH, VAS, PCS, and 15-D questionnaires. Radiographs will also be taken. Physical activity and the number of movements of the injured wrist will be evaluated with Axivity accelerometer in a subsample of patients. Operatively treated patients in Arm 2 will visit the clinic at 5 weeks and at 3 months, and the same follow-up protocol will be performed.

After the first week’s visit to the clinic for phase 2 patients, the same follow-up protocol as in the Cohort 1 will be used.

For all patients, PRWE will be the primary outcome and will be measured at the 1 and 2-year follow-up time points by the Internet, post, or telephone query. PRWE, PCS, pain, QuickDASH, 15-D, and self-assessment will be measured (Table 1). At the 1-year follow-up, physical activity and the number of wrist movements will be measured with Axivity accelerometer in a subgroup of patients. Grip strength will also be measured. At the 1- and 2-year follow-up, patient files will be reviewed to detect complications.

**Table 1:** Assessments and procedures

	Medical history	Radiograph	PRWE	EWC	PCS	Pain	Grip	Axivity	Quick-DASH	15-D	Self-assessment
Baseline	x	x		x	x					x	x
1 week (Cohort 2)	x	x									
5–6 weeks	(x)	x									
3 months	x	x	x		x	x	x	x	x	x	
1 year			x		x	x	x	x	x	x	x
2 years			x		x	x			x	x	x

### Radiological analysis

Standard radiological parameters will be defined from initial as well as final (3 months) radiographs. These parameters will be as follows: 1) volar-dorsal angulation angle, 2) the presence of dorsal and volar comminution, 3) radioulnar inclination angle, 4) ulnar variance 5) intra-articular step-off, 6) intra-articular diastasis. Two musculoskeletal radiologists will separately evaluate the radiographs. The radiologists will be blinded to the final treatment method and outcome while performing their assessment of the initial radiographs. From the initial radiographs, the probability given by EWC will be defined. To be pragmatic, we will not measure ulnar variance from the radiographs of the uninjured side as in the original article [28]. Instead, we suppose neutral variance.

### Power analysis

In this trial, a validated wrist specific PRWE-score will be used as the main outcome measure. In 2015, Wallenkamp and coworkers reported that the minimal clinically important difference in PRWE is 11 points and that SD is 14 [29]. Based on power calculations (Cl 95%, power 0.95, SD 14), the required sample size per group is 40 patients. Assuming a 30% drop-out rate based on possible surgical intervention during cast treatment, group size would be 57 per group (total 114). When two cohorts are taken into account, this would mean that a total of 228 patients is needed. Regardless of whether patients change to a different treatment group (cross-overs), the patients will be analyzed according to the intention-to-treat principle.

### Statistical analysis

The differences between groups in continuous skewed main outcome variables will be analyzed by the Mann-Whitney U-test, and analyzed by the t-test when variables are unskewed. The results will be presented with 95% confidence intervals. Two-way tables with the chi-square test will be used for dichotomous variables. In subgroup analysis, the effect of age, sex, fracture group, and smoking will be evaluated against the scores and overall quality of life after fracture.

Analysis of covariance will be used to assess the effect of pain catastrophizing score on the outcome of cast treatment. PRWE will be used as the dependent variable, cast as independent variable, and pain catastrophizing score as covariate.

The effect of cast immobilization treatment on the PRWE will also be investigated in the multivariate manner. Multivariable analysis will be performed with linear regression analysis since the outcome variable PRWE is normally distributed. The main variables of interest included will be cast immobilization and age, sex, fracture group, and smoking, and other diseases will be used as confounding variables. Self-assessment questionnaires (Additional files 1 and 2) will be analyzed and compared to find out which parameters are of the most importance for patients at the beginning of the treatment, and if the same parameters are equally important after the 1 and 2-year follow-up periods.

### Setting and recruitment

We need to recruit a total of 228 patients. Recruitment in Arm 3 will be challenging. As a single center, there is a risk that the required number of patients will not be achievable. This risk will be alleviated through Nordic collaboration by means of a multicenter study. When conducting a randomized controlled multicenter trial, the critical points are patient recruitment rate and key personnel stability. The collaboration between the different centers of the NITEP group has been found to be effective and reliable, and it has played crucial roles in the previous proximal humerus trial. This study will be carried out at Tampere University Hospital, Central Finland Central Hospital, Satakunta Central Hospital in Finland and Viborg regional Hospital in Denmark. We aim to maintain homogeneity between centers with solid trial designs, data, and project management including monitoring. Site personnel training has already started. We have been assisted by local research centers. The stability of the trial will be maintained with regular communications and bi-annual meetings.

### Data management

Each patient will be assigned a unique trial identification number (TIN) that will be matched with the patient’s identification number (ID). The matching key will be stored in a locked office at Tampere University Hospital, Finland, and the identification of each patient will only be possible after retrieving the matching key. Throughout the trial, the research data will be handled only with a TIN. The research data will be saved to a database with an online patient management program (PMP) located on a secure research server. The research data saved to the server will contain only anonymous TINs with a set of numbers acquired from the questionnaires, i.e., each question will be answered with a number. This will ensure the anonymity of each individual patient and that the identity of the patient will remain secret should the server data be revealed to third-parties.

All primary and secondary data will be acquired and stored on the study trial server. Data will be entered either by the patient during the control visits (via tablets) or by a researcher or study nurse when the questionnaires are mailed. The researchers from each hospital will have access to the secure study server where the trial research data is stored. The server has been approved by an information security committee at Tampere University Hospital. At the end of the trial, each researcher will have access to the data for further analyses. The questionnaires will be pre-programmed according to the PMP, and the individual patient data acquired at different time-points will be saved in Comma Separated Values (CSV) format, which is transferrable to, e.g., Microsoft Excel. All variables in the dataset will be described and suitable metadata standards will be used when available.

The copyright of the trial research data will be owned and created by the collaboration parties. The data will be shared freely among the collaboration parties. All participating researchers will receive access to the data after the trial is completed. Due to confidentiality and legal agreements, public data sharing will be restricted, (we have permission to store the data in the specific research server, but not to transfer the data). Under certain circumstances, e.g., when a new member joins the collaboration, we will grant access to the data. All data will be saved for 15 years after the end of the trial.

### Schedule

The patient recruitment will start in February 2018. The recruiting time will be 2 years and the results will be analyzed after a 1-year and a 2-year follow-up period.

## Discussion

Traditionally, the treatment decision (operative or non-operative) in fragility fractures has been based on the orthopaedic surgeon’s experience and beliefs. A few decades ago, this view was challenged by evidence-based medicine. As previously stated, a significant proportion of common orthopaedic interventions are not based on high-quality scientific evidence [10, 30]. Since the 2000s, randomized controlled trials (RCT) have become the gold standard in medical research.

We expect that an initial operative treatment of a DRF with volar locking plate will not yield superior results compared with a non-operative treatment with cast immobilization in terms of functional outcome, quality of life, disability, pain, grip strength, and number of complications among patients aged 65 and older. Moreover, we expect that functional outcome after treatment of distal radius fracture is related more to physical activity and catastrophic thinking than treatment modality. We assume that the late operative treatment of displaced distal radius fractures observed during short-term follow up will offer no additional benefit to continued non-operative treatment.

After our results are published, our aim will be to support the creation and dissemination of trustworthy guidelines by health authorities and professional organizations. We expect that after demonstrating the clinical results of our trial and supporting the creation and dissemination of trustworthy guidelines for the treatment policies of this common orthopaedic complaint, Nordic countries will be substantially changed and will reflect the best current evidence.

## Additional files

Additional file 1: Patient self-assessment – initial situation. (DOCX 101 kb)

Additional file 2: Patient self-assessment – 1 year, 2 years. (DOCX 101 kb)

## Abbreviations

15-D: 15-dimensional
CI: Confidence interval
CSV: Comma separated values
DRF: Distal radius fracture
ER: Emergency room
EWC: Edinburgh wrist calculator
ID: Identification
NITEP: Nordic innovative trial to evaluate osteoporotic fractures
PASS: Patient self-assessed state of symptom
PCS: Pain catastrophizing score
PMP: Patient management program
PROM: Patient rated outcome measure
PRWE: Patient rated wrist evaluation
Quick-DASH: Quick- disabilities of arm, shoulder and hand
SD: Standard deviation
TIN: Trial identification number
VAS: Visual analogue scale

## References

[1] Lauritzen JB, Schwarz P, Lund B, McNair P, Transbol I (1993). Changing incidence and residual lifetime risk of common osteoporosis-related fractures. Osteoporos Int.

[2] Seeley DG, Browner WS, Nevitt MC, Genant HK, Scott JC, Cummings SR (1991). Which fractures are associated with low appendicular bone mass in elderly women? The study of osteoporotic fractures research group. Ann Intern Med.

[3] Court-Brown CM, Caesar B (2006). Epidemiology of adult fractures: a review. Injury.

[4] Larsen CF, Lauritsen J (1993). Epidemiology of acute wrist trauma. Int J Epidemiol.

[5] Flinkkila T, Sirnio K, Hippi M, Hartonen S, Ruuhela R, Ohtonen P, Hyvonen P, Leppilahti J. Epidemiology and seasonal variation of distal radius fractures in Oulu, Finland. Osteoporos Int. 2011; 10.1007/s00198-010-1463-3. 10.1007/s00198-010-1463-3 20972668

[6] Brogren E, Petranek M, Atroshi I (2007). Incidence and characteristics of distal radius fractures in a southern Swedish region. BMC Musculoskelet Disord.

[7] Shauver MJ, Clapham PJ, Chung KC. An economic analysis of outcomes and complications of treating distal radius fractures in the elderly. J Hand Surg Am. 2011; 10.1016/j.jhsa.2011.09.039. 10.1016/j.jhsa.2011.09.039 22123045

[8] Pirkanmaan sairaanhoitopiiri. Tuotehinnasto. 2017. http://www.pshp.fi/download/noname/%7B2F6BDDB9-65DF-4F6A-81D0-F957E1D2737A%7D/51137.

[9] Helsingin ja Uudenmaan sairaanhoitopiiri. Palveluhinnasto. 2018. http://www.hus.fi/hus-tietoa/talous/Hinnoittelu/Documents/HUS%20Palveluhinnasto%202018.pdf.

[10] Lohmander LS, Roos EM. The evidence base for orthopaedics and sports medicine. BMJ. 2015; 10.1136/bmj.g7835. 10.1136/bmj.g7835 25555826

[11] Mattila VM, Huttunen TT, Sillanpaa P, Niemi S, Pihlajamaki H, Kannus P. Significant change in the surgical treatment of distal radius fractures: a nationwide study between 1998 and 2008 in Finland. J Trauma. 2011; 10.1097/TA.0b013e3182231af9. 10.1097/TA.0b013e3182231af9 21986738

[12] Wilcke MKT, Hammarberg H, Adolphson PY. Epidemiology and changed surgical treatment methods for fractures of the distal radius: a registry analysis of 42,583 patients in Stockholm County, Sweden, 2004-2010. Acta Orthop. 2013; 10.3109/17453674.2013.792035. 10.3109/17453674.2013.792035 PMC3715813 23594225

[13] Tarallo L, Mugnai R, Adani R, Catani F (2014). Malunited extra-articular distal radius fractures: corrective osteotomies using volar locking plate. J Orthop Traumatol.

[14] Esposito J, Schemitsch EH, Saccone M, Sternheim A, Kuzyk PRT. External fixation versus open reduction with plate fixation for distal radius fractures: a meta-analysis of randomised controlled trials. Injury. 2013; 10.1016/j.injury.2012.12.003. 10.1016/j.injury.2012.12.003 23298757

[15] Costa ML, Achten J, Parsons NR, Rangan A, Griffin D, Tubeuf S, Lamb SE, DRAFFT Study Group. Percutaneous fixation with Kirschner wires versus volar locking plate fixation in adults with dorsally displaced fracture of distal radius: randomised controlled trial. BMJ. 2014; 10.1136/bmj.g4807. 10.1136/bmj.g4807 PMC4122170 25096595

[16] Grewal R, MacDermid JC (2007). The risk of adverse outcomes in extra-articular distal radius fractures is increased with malalignment in patients of all ages but mitigated in older patients. J Hand Surg [Am].

[17] Wei J, Yang T, Luo W, Qin J, Kong F. Complications following dorsal versus volar plate fixation of distal radius fracture: a meta-analysis. J Int Med Res. 2013; 10.1177/0300060513476438. 10.1177/0300060513476438 23569022

[18] Leung F, Ozkan M, Chow SP (2000). Conservative treatment of intra-articular fractures of the distal radius--factors affecting functional outcome. Hand Surg.

[19] Plant CE, Parsons NR, Costa ML. Do radiological and functional outcomes correlate for fractures of the distal radius? Bone Joint J. 2017; 10.1302/0301-620X.99B3.35819. 10.1302/0301-620X.99B3.35819 28249979

[20] Kodama N, Takemura Y, Ueba H, Imai S, Matsusue Y. Acceptable parameters for alignment of distal radius fracture with conservative treatment in elderly patients. J Orthop Sci. 2014; 10.1007/s00776-013-0514-y. 10.1007/s00776-013-0514-y 24338051

[21] Martinez-Mendez D, Lizaur-Utrilla A, de-Juan-Herrero J. Intra-articular distal radius fractures in elderly: a randomized prospective study of casting versus volar plating. J Hand Surg Eur Vol. 2017; 10.1177/1753193417727139. 10.1177/1753193417727139 28870129

[22] Jiang Y, Sanchez-Santos MT, Judge AD, Murray DW, Arden NK (2017). Predictors of patient-reported pain and functional outcomes over 10 years after primary Total knee arthroplasty: a prospective cohort study. J Arthroplast.

[23] Sullivan MJL, Bishop SR, Pivik J (1995). The pain catastrophizing scale: development and validation. Psychol Assess.

[24] Teunis T, Bot AGJ, Thornton ER, Ring D. Catastrophic thinking is associated with finger stiffness after distal radius fracture surgery. J Orthop Trauma. 2015; 10.1097/BOT.0000000000000342. 10.1097/BOT.0000000000000342 25866942

[25] Arora R, Lutz M, Deml C, Krappinger D, Haug L, Gabl M. A prospective randomized trial comparing nonoperative treatment with volar locking plate fixation for displaced and unstable distal radial fractures in patients sixty-five years of age and older. J Bone Joint Surg Am. 2011; 10.2106/JBJS.J.01597. 10.2106/JBJS.J.01597 22159849

[26] Anzarut A, Johnson JA, Rowe BH, Lambert RGW, Blitz S, Majumdar SR (2004). Radiologic and patient-reported functional outcomes in an elderly cohort with conservatively treated distal radius fractures. J Hand Surg [Am].

[27] Batra S, Gupta A (2002). The effect of fracture-related factors on the functional outcome at 1 year in distal radius fractures. Injury.

[28] Mackenney PJ, McQueen MM, Elton R (2006). Prediction of instability in distal radial fractures. J Bone Joint Surg Am.

[29] Walenkamp MMJ, de Muinck KR, Goslings JC, Vos LM, Rosenwasser MP, Schep NWL. The minimum clinically important difference of the patient-rated wrist evaluation score for patients with distal radius fractures. Clin Orthop. 2015; 10.1007/s11999-015-4376-9. 10.1007/s11999-015-4376-9 PMC4562929 26040969

[30] Lim HC, Adie S, Naylor JM, Harris IA. Randomised trial support for orthopaedic surgical procedures. PLoS One. 2014; 10.1371/journal.pone.0096745. 10.1371/journal.pone.0096745 PMC4057075 24927114

