# Supplementary material for: Nordic Innovative Trial to Evaluate OsteoPorotic Fractures (NITEP-group): non-operative treatment versus surgery with volar locking plate in the treatment of distal radius fracture in patients aged 65 and over – a study protocol for a prospective, randomized controlled trial
Source: BMC Musculoskelet Disord. 2018 Apr 5;19:106. doi: 10.1186/s12891-018-2019-5 (PMC5887252; doi:10.1186/s12891-018-2019-5)
Supplement: Supplementary file 2 — Patient self-assessment – 1 year, 2 years. (DOCX 101 kb) [file 12891_2018_2019_MOESM2_ESM.docx]

**Patient self-assessment – 1 year, 2 years Date: 21.5.2017 (ver 1.0) Researcher: Antti Launonen**

**Answer the following statements and questions. Circle the most appropriate answer/number.**

|  | Completely disagree | Disagree | Neither agree or disagree | Agree | Completely agree |
| --- | --- | --- | --- | --- | --- |
| It is important for me that there is no pain in the wrist after the treatment has ended. | 1 | 2 | 3 | 4 | 5 |

|  | Completely disagree | Disagree | Neither agree or disagree | Agree | Completely agree |
| --- | --- | --- | --- | --- | --- |
| It is important for me that after the treatment my wrist operates the same way it did before the fracture. | 1 | 2 | 3 | 4 | 5 |

|  | Completely disagree | Disagree | Neither agree or disagree | Agree | Completely agree |
| --- | --- | --- | --- | --- | --- |
| It is important for me that after the treatment my wrist looks the same way it did before the fracture. | 1 | 2 | 3 | 4 | 5 |

|  | Completely disagree | Disagree | Neither agree or disagree | Agree | Completely agree |
| --- | --- | --- | --- | --- | --- |
| I believe that rehabilitation will have a more significant impact on the end result than whether I undergo surgery or not. | 1 | 2 | 3 | 4 | 5 |

|  | Completely disagree | | Disagree | Neither agree or disagree | Agree | Completely agree |
| --- | --- | --- | --- | --- | --- | --- |
| It is important for me to understand what is being done to me and why. | | 1 | 2 | 3 | 4 | 5 |

**1. Are you satisfied with the treatment you have received?**

**YES NO**

**2. Are you satisfied with how your wrist functions at the moment?**

**YES NO**

**3. Are you satisfied with participating in this study?**

**YES NO**

**4. Do you wish that chance had assigned you another treatment option?**

**YES NO**
